# An Ecological Framework for Interpreting the Canine Gut Microbiome

**DOI:** 10.3390/ani16121787

**Published:** 2026-06-09

**Authors:** Bernard Walther, Fabrice Bouilloux, Philippe Vayer, Alexandre Douablin, Fanny Walther

**Affiliations:** 1 AREGTeC, 12 Rue des Ormeaux, 45150 Darvoy, France; philippe.vayer@gmail.com (P.V.); walther.fanny@gmail.com (F.W.); 2 Biomnigene, 48 Rue des Founottes, 25000 Besançon, France; bouilloux.fabrice@biomnigene.com (F.B.); douablin.alexandre@biomnigene.com (A.D.)

**Keywords:** canine microbiome, fecal calprotectin, dysbiosis, microbiome resilience, microbiological inflammatory score, gut ecology, 16S rRNA

## Abstract

The intestinal microbiome is increasingly recognized as an important determinant of canine gastrointestinal health. However, interpreting microbiome sequencing data remains challenging because most analytical approaches rely on taxonomic descriptions, alpha diversity indices, or dysbiosis indices derived generally from a limited number of microbial ecological interpretation targets. While shotgun metagenomic approaches increasingly allow the identification of microbial communities, such analyses remain costly and are not yet widely accessible in routine veterinary settings. The objective of this study was to develop an integrative interpretation framework based on widely accessible biomarkers combining fecal calprotectin and 16S rRNA gene sequencing data. These data enabled the generation of complementary ecological dimensions of gut microbiome organization: biological inflammation assessed through fecal calprotectin, microbiological inflammatory pressure estimated through a Microbiological Inflammatory Score (MIS), and microbiome stability measured by a Microbiome Resilience Score (MRS) derived from alpha diversity, functional balance, and dominance structure. Fecal microbiome profiles obtained by 16S rRNA gene sequencing were analyzed in a real-life cohort of privately owned dogs. Alpha diversity, taxonomic weighting, abundance-dependent dominance rules, beta diversity based on Bray–Curtis dissimilarity, distance to a reference microbiome core, and a 16S-derived dysbiosis score were integrated into a multidimensional interpretation model. Strong ecological associations were observed between resilience, microbial diversity, and dysbiosis-related metrics. Microbiome resilience strongly correlated with Shannon diversity (Spearman ρ = 0.98, *p* < 0.001), while the reconstructed 16S-derived dysbiosis score showed a more moderate positive correlation with MIS (Spearman ρ = 0.41, *p* = 0.004), supporting the partially independent ecological dimensions captured by the framework. The results revealed a continuum ranging from stable microbiomes to inflammatory dysbiosis. Most dogs clustered near a reference microbiome core characterized by low microbiological inflammatory pressure and high resilience, whereas a subset of microbiomes showed elevated MIS values, reduced resilience, increased compositional distance from the reference core, and higher dysbiosis index values. These findings support the value of a multidimensional experimental framework integrating inflammation, dysbiosis, and resilience to improve interpretation of canine microbiome profiles under real-life conditions.

## Simple Summary

Understanding a complex ecosystem such as the intestinal microbiome is of importance in canine health. Using analysis of fecal samples from a diverse cohort of dogs, we developed an integrated framework to assess the intestinal microbiome status using biological inflammation (fecal calprotectin levels), microbial imbalance (Microbiological Inflammatory Score, MIS), and ecological stability (resilience and core bacterial taxa). Fecal calprotectin reflects host inflammatory response, but does not necessarily describe microbial structure. The MIS gives information on the changes in the microbiome from stability to dysbiosis and the resilience indicates the system’s capacity to cope with disturbances. This approach illustrates how microbiome configurations range from resilient and balanced communities through transitional to structurally fragile states. The combination of biological markers with the ecological status of the microbiome provides a more comprehensive and operational view of canine gut health and may contribute to improved interpretation of microbiome data in veterinary practice.

## 1. Introduction

The canine gut microbiome is increasingly recognized as an important component of gastrointestinal health and disease. 16S rRNA gene sequencing has enabled detailed characterization of intestinal microbial communities in dogs [1,2,3,4]. They contribute to multiple physiological processes including nutrient metabolism, immune regulation, epithelial barrier maintenance, bile acid transformation, and resistance to colonization by pathogenic organisms [1,2,3,4].

Alterations in microbiome composition have been reported in a variety of gastrointestinal disorders in dogs, including acute diarrhea, chronic inflammatory enteropathies, antibiotic-associated dysbiosis, and dietary transitions [5,6,7,8,9,10,11,12,13,14,15,16].

Dogs with chronic enteropathies often exhibit reduced bacterial diversity and shifts in several key taxa such as Faecalibacterium, Blautia, Turicibacter, and members of Fusobacterium and Proteobacteria [5,6,7,8]. Antibiotic exposure, particularly to metronidazole or tylosin, can also induce significant changes in the intestinal microbiome that may persist beyond the treatment period [13,14,15,16].

Despite these advances, translating microbiome sequencing data into more meaningful interpretation remains difficult. Many current analytical approaches rely on a limited set of descriptors. Alpha diversity metrics such as richness and Shannon diversity provide information on community complexity but do not capture functional imbalance or inflammatory potential. A dysbiosis index, often based on quantitative PCR of selected bacterial taxa, can detect deviations from a healthy reference profile but provide only a partial view of microbiome organization [7,8]. As a result, microbiome profiles may remain difficult to understand in clinical or research contexts.

Another important consideration is that microbial alterations and inflammatory responses do not necessarily occur in parallel. This partial dissociation complicates interpretation of microbiome data in clinical settings. Changes in microbiome composition may occur without detectable inflammatory biomarkers, whereas intestinal inflammation can sometimes occur, despite relatively preserved microbial diversity [6,17,18,19]. This complicates the interpretation of microbiome data and raises questions about the relationship between dysbiosis and intestinal inflammatory activity.

Fecal calprotectin has been described as a valuable non-invasive biomarker of intestinal inflammation in dogs [17,18,19]. Released by activated neutrophils and monocytes, this protein reflects neutrophil-associated mucosal inflammation and provides an objective measure of intestinal inflammatory activity, but cannot alone characterize the status of the intestinal microbial ecosystem. Chronic inflammatory enteropathies are currently recognized as a heterogeneous group of disorders with evolving clinical classifications [20].

Diet and environmental exposure also play a major role in shaping the canine gut microbiome. Distinct microbial profiles have been associated with different feeding practices such as commercial kibble diets, fresh diets, and raw feeding [10,11,12]. However, microbiomes with apparently similar composition may differ markedly in their ecological stability and resilience. Beyond taxonomic composition alone, ecological concepts such as resilience and ecosystem stability, as well as microbiome-derived biomarkers including the canine Dysbiosis Index, have increasingly contributed to the interpretation of canine microbiome profiles [21,22,23,24]. The objective of this exploratory study was therefore to develop an interpretation framework integrating three complementary dimensions of canine gut microbiome status: intestinal inflammatory activity assessed by fecal calprotectin, microbiological imbalance quantified through a Microbiological Inflammatory Score, and microbiome stability quantified through a Microbiome Resilience Score. By combining inflammatory biomarkers with microbiome-derived metrics, an approach increasingly advocated for microbiome-based biomarkers [25], this framework aims to provide a structured approach for interpreting canine microbiome profiles under real-life conditions.

## 2. Materials and Methods

### 2.1. Study Population and Recruitment

The cohort consisted of privately owned dogs recruited under real-life conditions for fecal microbiome analysis. This recruitment strategy was designed to preserve ecological diversity within the cohort while improving the reliability of owner-reported information. More than 300 applications were received. Prior to inclusion, dogs were categorized according to age (<8 years or ≥8 years) and health-related questionnaire information in order to ensure representation across age groups and clinical conditions. Categories included healthy controls and dogs presenting digestive disorders, other medical conditions, osteoarthritis, or recent medication exposure, particularly antibiotics, anti-inflammatory drugs, and anticancer treatments. The initial recruitment target was approximately 50 dogs distributed across these categories. Following verification of questionnaire data, sample eligibility assessment, and owner withdrawals, the final study population consisted of 45 dogs. Inflammatory status and microbiome results were unknown at the time of recruitment and therefore did not influence inclusion.

From the initial applicants, 45 dogs were selected for inclusion. Health information provided by the owners was verified through structured telephone interviews. Owners received a written protocol together with a video demonstration to ensure standardized sample collection. This recruitment and verification procedure was designed to ensure both reliability of the collected data and diversity within the study population.

Extensive metadata including dietary information, supplementation, medication exposure, breed, age, and environmental variables were collected through owner questionnaires prior to sample collection. However, these variables were not systematically integrated into the present analyses because the primary objective of this study was first to establish the internal ecological coherence of the proposed multidimensional microbiome framework.

### 2.2. Sample Inclusion and Dataset Structure

A total of 45 dogs were included in the main analysis dataset. Two additional microbiome profiles (profiles 46 and 47 in the internal dataset) corresponded to repeated samples obtained due to delayed reception or substitution of calprotectin-associated material in two animals.

These repeated profiles were retained for comparative 16S analyses but were excluded from the integrated analysis in order to avoid pseudo-replication and preserve statistical independence between samples. Consequently, the merged abundance matrix contained 47 microbiome profiles, whereas all integrated analyses and publication numbering were based on 45 unique dogs.

Throughout the manuscript, dogs are referred to by their order number in the cohort results table.

### 2.3. Sample Collection

Fecal samples intended for calprotectin measurement were collected in clean, dry containers without detergent or stabilizing agents. Analyses were performed upon reception of the samples, with a maximum storage time of eight days at room temperature.

Samples intended for microbiome sequencing were collected separately and immediately stored in SLX-Mus buffer (Omega Bio-tek, Norcross, GA, USA, Ref: SLXMLUS-1000) until DNA extraction.

### 2.4. Calprotectin Measurement

Fecal calprotectin concentrations were used as a biological marker of intestinal inflammatory activity [17]. Concentrations were measured using a human colloidal gold immunochromatographic assay (CALPROTECTIN-CHECK-1 Quantitative determination of calprotectin in faeces samples FOR EASY READER^®^ AND EASY READER+^®^ USE ONLY Ref. 63091 (20 tests)/Ref. 63091-10T (10 tests), Manufactured by VEDALAB—Cerisé, France) adapted for canine fecal samples.

For interpretation, calprotectin values were categorized as follows:

Absence of inflammation ≤ 6.25 µg/g (BLQ/2);
Low inflammatory activity > 6.25 to ≤19 µg/g;
Moderate inflammatory activity ≥ 19 to 50 µg/g;
High inflammatory activity > 50 µg/g [17,18,19].

These categories were used as contextual biological descriptors in the interpretation framework.

The fecal calprotectin assay was selected because it provides a rapid and accessible quantification of biological inflammatory activity compatible with animal sample evaluation. Its use in canine samples was supported by the biological conservation and interspecies homology of calprotectin proteins (S100A8/S100A9) between humans and dogs and by previous veterinary studies evaluating fecal calprotectin measurement in dogs. Concentration controls were performed before sample measurements according to the manufacturer’s procedure.

### 2.5. DNA Extraction and 16S rRNA Sequencing

Microbial community structure was characterized using 16S rRNA gene sequencing. DNA extraction followed Biomnigene’s standardized protocol for nucleic acid isolation. PCR amplification targeted the V3–V4 hypervariable regions of the bacterial 16S rRNA gene using primers designed to amplify a fragment of approximately 420 bp.

Amplicons were pooled and sequenced on an Illumina MiSeq platform using a 2 × 251 bp paired-end protocol.

### 2.6. Bioinformatic Processing

Sequencing reads were demultiplexed using cutadapt (v5.0), and quality control was assessed using FastQC (v0.11.9) and MultiQC. Adapter trimming and quality filtering were performed using fastp (v0.23.2).

Bioinformatic analyses were conducted with QIIME2 (v2025.10.1). Primer sequences were removed using the cutadapt plugin, followed by denoising, chimera removal, and paired-end merging using the DADA2 plugin implemented in QIIME2. Reads with two or more predicted sequencing errors based on quality scores were discarded during denoising. Paired-end reads were merged using a minimum overlap of 8 bp.

Sequencing and denoising resulted in satisfactory sequencing depth and quality across samples, with a mean sequencing depth of approximately 8110 raw reads per sample and an average of 5517 non-chimeric reads retained after processing. Approximately 69% of reads were retained after filtering and chimera removal.

Taxonomic assignment was performed against the GTDB reference database (release 220) using the classify-sklearn method. Alpha and beta diversity metrics were calculated within QIIME2, with Bray–Curtis dissimilarity used as the principal distance metric.

All downstream analyses were performed on relative abundance data rather than rarefied counts. This approach was selected to limit information loss associated with rarefaction procedures and to preserve ecological information across samples.

Because microbiome sequencing data are compositional by nature, ecological interpretations based on relative abundance should be interpreted cautiously and primarily as comparative ecosystem-level descriptors rather than direct quantitative measurements of bacterial biomass. Bray–Curtis dissimilarity was selected because it is widely used in microbial ecology for abundance-sensitive comparison of community structure and ecological divergence between microbiome configurations.

A minimum detection threshold of 0.1% relative abundance was applied for inclusion of taxa in functional scoring. Relative abundance filtering and normalization were applied consistently across all downstream ecological analyses.

### 2.7. Abundance Matrix Reconstruction

Because the cohort originated from two partially distinct abundance files, a merged abundance matrix was reconstructed. The first abundance file corresponded to the first 11 dogs, whereas the second contained the remaining 35 dogs together with additional taxonomic detail.

The two matrices were harmonized taxonomically across levels 1 to 7, sample identifiers were standardized, and absent taxa were assigned a value of zero. This process produced a single operational abundance matrix used for downstream analyses.

The main calculations were performed at genus level (level 6), while species-level information was retained for selected dysbiosis-associated taxa.

### 2.8. Microbiological Inflammatory Score (MIS)

The Microbiological Inflammatory Score (MIS) quantifies the inflammatory pressure associated with microbiome composition. The score was operationally calculated as the weighted sum of taxon abundances based on their assigned functional roles.

Taxa were operationally classified as protective, neutral, pro-inflammatory, or context-dependent according to a curated database derived from canine microbiome literature [1,2,3,4,5,6,7,8]. Protective taxa received negative weights, pro-inflammatory taxa positive weights. Context-dependent taxa were handled through abundance-dependent dominance rules because their ecological interpretation may vary according to relative abundance and community structure.

To facilitate comparison inter-individual comparison, MIS values were normalized relative to a reference subset composed of dogs with absent or low calprotectin values and preserved microbiome structure. The median absolute raw MIS value observed in this subset was used as normalization factor. Because the present study was exploratory and cohort-based, the weighting scheme and thresholds used for MIS calculation should be considered cohort-specific operational interpretative parameters rather than externally validated diagnostic cut-offs. Similarly, the proposed ecological framework should currently be interpreted as a hypothesis-generating microbiome interpretation model whose reproducibility and portability remain to be evaluated in independent canine cohorts.

Scaled MIS values were interpreted as follows: ≤1 low inflammatory pressure, 1–3 mild imbalance, 3–6 moderate dysbiosis, >6 high dysbiotic configurations.

These thresholds represent exploratory ecological interpretation categories rather than externally validated diagnostic cut-offs.

### 2.9. Dominance Rules

Dominance adjustments were introduced to account for abundance-dependent ecological destabilization rather than direct inflammatory activity. Some taxa exhibit context-dependent behavior depending on their abundance within the microbial community. Two operational thresholds were defined:

T1 corresponding to the onset of ecological dominance;
T2 corresponding to strong ecological dominance.

Thresholds used in this cohort were:

Megamonas: 15% and 25%; Fusobacterium: 10% and 20%; Enterobacteriaceae: 2% and 5%; Proteus: 1% and 3%.

These thresholds were empirically derived from abundance distributions observed within the cohort together with previously reported ecological patterns in canine dysbiosis studies. Their objective was not to define rigid biological cut-offs, but rather to identify abundance ranges associated with progressive ecological dominance and potential ecosystem destabilization.

Because the ecological interpretation of certain taxa may vary according to relative abundance and microbial community structure, taxa such as Fusobacterium and Megamonas were treated as context-dependent ecological taxa rather than systematically classified as inflammatory organisms.

Above T2, the taxon was interpreted as a potential destabilizing ecological driver. Formal threshold sensitivity analyses were not performed in the present study and these operational thresholds should therefore be interpreted cautiously.

### 2.10. Microbiome Resilience Score (MRS)

The Microbiome Resilience Score (MRS) provides an ecological estimate of microbiome structural stability and resilience. The score should be interpreted as an exploratory ecological descriptor rather than as a validated clinical biomarker. The score integrates four components: normalized alpha diversity, normalized evenness, functional balance between protective and pro-inflammatory taxa, a dominance penalty.

The raw score ranges from 0 to 1. A cubic transformation was applied only for selected graphical representations to improve visual separation of highly resilient microbiomes. Raw values were retained for interpretation and in the results table.

### 2.11. Beta Diversity and Reference Microbiome Core

Between-sample distances were calculated using Bray–Curtis dissimilarity on the merged genus-level abundance matrix.

An ecological reference microbiome core was defined using dogs presenting low calprotectin levels, low MIS values, preserved diversity, and absence of marked pathobiont dominance. In the final dataset this core corresponded to Dog 15, Dog 22, Dog 25, Dog 28, Dog 29, and Dog 44.

Distances reported in the analyses correspond to Bray–Curtis dissimilarity between each microbiome and the centroid derived from this reference core.

Additional methodological details regarding the calculation of MIS, MRS, alpha and beta diversity metrics, reference core microbiome construction, and the ecological interpretation framework are provided in Supplementary File S1.

### 2.12. Dysbiosis Index Comparison

For comparison with existing canine microbiome literature, an approximate 16S-derived dysbiosis score was reconstructed from taxa commonly associated with dysbiotic states [7,8,24]. The score was derived from relative abundance profiles obtained by 16S rRNA gene sequencing and integrated the balance between taxa commonly associated with microbiome stability and taxa frequently enriched in dysbiotic configurations. Protective taxa included Faecalibacterium, Blautia, Turicibacter, and Peptacetobacter hiranonis. Pathobiont-associated taxa included Streptococcus, Proteus, Fusobacterium, Clostridium groups, and Enterobacteriaceae.

Although conceptually inspired by the canine dysbiosis index proposed by AlShawaqfeh et al. [7], the score used here differs methodologically because it is derived from relative abundance profiles rather than absolute qPCR quantification of seven targeted taxa. Consequently, it should therefore be interpreted as an exploratory ecological descriptor rather than as a diagnostic threshold.

Conceptual interpretation of the canine gut microbiome.

**Figure 1:**
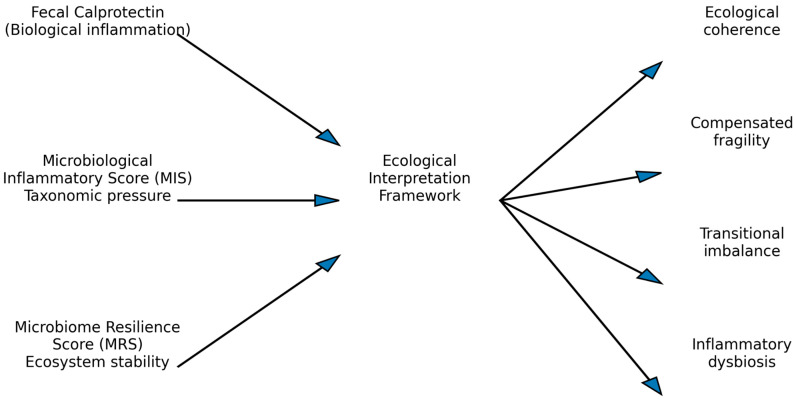
Conceptual interpretation of the canine gut microbiome.

The model, described in Figure 1 integrates biological inflammation assessed by fecal calprotectin, microbiological inflammatory pressure assessed by MIS, microbiome resilience, ecological distance derived from Bray–Curtis dissimilarity, and a 16S-derived dysbiosis score.

### 2.13. Statistical Analysis

Statistical analyses were performed using R software (R Foundation for Statistical Computing, Vienna, Austria; version 4.3 or later). Data handling and graphical exploration were performed using the tidyverse package. Ecological analyses were performed using the vegan package.

Because microbiome-derived ecological variables are not necessarily normally distributed and because of the moderate cohort size, non-parametric statistics were prioritized. Associations between ecological variables, including fecal calprotectin, MIS, MRS, Bray–Curtis distance, and the reconstructed 16S-derived dysbiosis score, were primarily assessed using Spearman’s rank correlation coefficient. Pearson correlation coefficients were additionally calculated for graphical comparison when linear ecological relationships were explored.

Group comparisons were performed using Wilcoxon rank-sum tests or Kruskal–Wallis tests where appropriate. When multiple taxonomic comparisons were performed, *p*-values were adjusted using the Benjamini–Hochberg false discovery rate correction.

Because of the exploratory ecological nature of the study and the limited size of some inflammatory subgroups, statistical analyses were interpreted primarily as ecological associations rather than inferential diagnostic validation. Exact *p*-values were reported where appropriate, and a *p*-value < 0.05 was considered statistically significant.

## 3. Results

The analysis included 45 dogs with complete microbiome and calprotectin data. Calprotectin classes were distributed as follows: 21 dogs with absent inflammatory signal, 14 with low inflammatory activity, 6 with moderate inflammatory activity, and 4 with high inflammatory activity. This distribution indicates that the cohort covered a broad range of inflammatory status while remaining dominated by dogs with absent or limited fecal inflammatory activity.

This distribution provided sufficient ecological variability across the cohort to explore relationships between inflammatory status and microbiome ecological structure while preserving a large subset of microbiomes consistent with physiological stability. The number of dogs presenting high inflammatory activity remained limited within the cohort. This limits subgroup-level inference for this phenotype.

The sequencing workflow yielded satisfactory read depth and sequence quality across samples after denoising and chimera filtering.

Substantial variability was observed across the cohort in microbiome diversity, inflammatory pressure, resilience, compositional distance, and dysbiosis index values. Shannon diversity ranged from 1.04 to 3.56, Pielou evenness from 0.53 to 0.94, MIS_scaled from 0.00 to 3.58, MRS from 0.33 to 0.97, Bray–Curtis distance to the reference core from 0.372 to 0.951, and the 16S-derived dysbiosis score (not directly comparable to the qPCR-based canine dysbiosis index) from −3.380 to 19.066. These ranges indicate substantial heterogeneity in microbiome structure across the cohort, spanning communities close to the reference configuration to markedly altered microbiomes.

Alpha diversity varied widely across the cohort, from highly diverse and evenly distributed communities to simplified microbiomes dominated by a limited number of taxa. Shannon diversity and Pielou evenness were strongly associated with the resilience score, with higher resilience values observed in microbiomes displaying both high diversity and balanced taxonomic structure (Table 1). The strongest association observed within the cohort involved microbiome resilience and alpha diversity metrics. MRS strongly correlated with Shannon diversity (Spearman ρ = 0.98; *p* < 0.001), with linear regression analysis also demonstrating a strong association (R^2^ = 0.82; Pearson r = 0.91). However, diversity alone did not fully characterize microbiome organisation, as some communities with intermediate diversity displayed reduced resilience because of dominance patterns or unfavorable functional balance. More moderate associations were observed between dysbiosis-related metrics and microbiological inflammatory pressure. The reconstructed 16S-derived dysbiosis score positively correlated with MIS (Spearman ρ = 0.41, *p* = 0.004), whereas microbiome resilience negatively correlated with dysbiosis severity (Spearman ρ = −0.51, *p* < 0.001). This moderate correlation suggests that dysbiosis severity and microbiota-associated inflammatory signatures represent related but partially independent ecological dimensions within the cohort.

The Microbiological Inflammatory Score showed a heterogeneous distribution across the cohort. Most dogs exhibited low to moderate MIS values, consistent with limited microbiological inflammatory pressure within the cohort.

Microbiological inflammatory pressure reflects the relative enrichment of bacterial taxa commonly associated with dysbiotic or inflammatory gut environments described in canine enteropathies.

The Microbiome Resilience Score also showed substantial variability across the cohort. High resilience values were observed primarily in microbiomes characterized by high diversity, preserved evenness, balanced functional composition, and absence of strong dominance by potentially inflammatory taxa. In contrast, low resilience values were associated with simplified or dominance-driven microbiomes.

In the present framework, resilience represents an operational ecological descriptor derived from diversity and compositional balance rather than a directly measured functional property. Associations between ecological metrics were evaluated using both Pearson and Spearman correlation analyses. Microbiome resilience also remained strongly associated with alpha diversity metrics across the cohort. A weaker negative association was observed between MRS and MIS (r ≈ −0.30), indicating that inflammatory pressure and structural resilience represent partially independent ecological dimensions.

**Figure 2:**
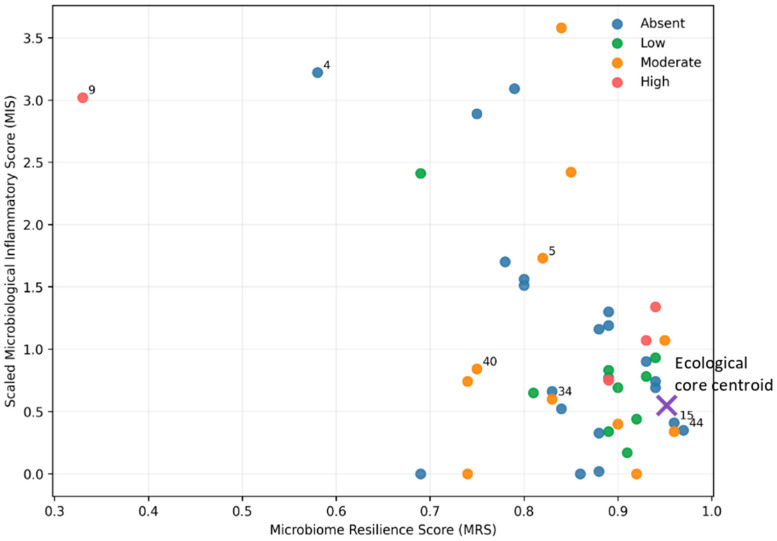
Positioning of canine microbiomes in MIS–MRS space. Each point represents one dog. The x-axis corresponds to the Microbiome Resilience Score (MRS), and the y-axis corresponds to the scaled Microbiological Inflammatory Score (MIS_scaled). Inflammatory categories were assigned according to fecal calprotectin concentrations as described in Section 2.4: absence of inflammation (≤6.25 µg/g), low inflammatory activity (>6.25–19 µg/g), moderate inflammatory activity (≥19–50 µg/g), and high inflammatory activity (>50 µg/g). The ordination illustrates substantial overlap between inflammatory categories, indicating that inflammatory activity and microbiome ecological structure are related but not fully concordant. The ecological reference core centroid is represented by a purple cross.

The MIS–MRS representation illustrated a continuum of microbiome configurations ranging from stable communities to transitional imbalance, compensated fragility, and dysbiosis-associated state (Figure 2).

The centroid of the ecological reference core was calculated from microbiomes meeting predefined stability criteria and used as a spatial reference for positioning individual microbiomes along this ecological gradient.

The combined MIS–MRS representation, together with calprotectin and beta diversity descriptors, highlighted several representative microbiome configurations within the cohort, illustrated by selected individual profiles. Dog 44 illustrated a configuration characterized by preserved diversity, low MIS, and high resilience without detectable inflammatory activation. Dog 15 represented one of the most resilient microbiome profiles observed in the cohort, combining low inflammatory pressure, high diversity, and strong structural stability. Dog 41 also corresponded to a stable configuration with preserved microbiome structure and limited inflammatory pressure. Dogs 34 and 40 represented intermediate configurations characterized by moderate inflammatory pressure and partially preserved resilience, whereas Dog 5 illustrated a configuration with moderate biological inflammation and moderately increased microbiological inflammatory pressure despite partially preserved resilience. To further illustrate the model, Dog 4 represented a state of compensated fragility characterized by reduced diversity and resilience in the absence of marked inflammation, while Dog 9 corresponded to a configuration compatible with inflammatory dysbiosis, combining high calprotectin levels, elevated inflammatory pressure, increased distance from the reference core, and reduced resilience.

These representative profiles illustrate the diversity of microbiome configurations observed across the MIS–MRS ecological space.

**Figure 3:**
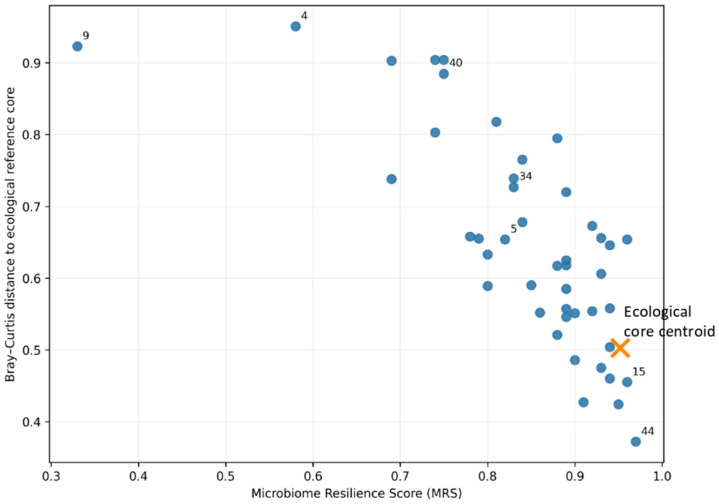
Beta diversity structure of the canine microbiome cohort. Between-sample dissimilarity was quantified using Bray–Curtis distance. The highlighted reference core corresponds to microbiomes characterized by low calprotectin, low microbiological inflammatory pressure, preserved diversity, and absence of marked pathobiont dominance. Distances correspond to Bray–Curtis dissimilarity between each microbiome and the centroid derived from the ecological reference core.

Beta diversity analysis based on Bray–Curtis distances revealed a structured organization of the cohort (Figure 3). Microbiomes characterized by low inflammatory pressure and high resilience clustered around the reference core. These microbiomes combine low inflammatory pressure, preserved diversity, and strong resilience, forming a coherent ecological reference subset within the cohort. In contrast, dysbiotic or structurally fragile microbiomes occupied more distant positions.

This spatial structure indicates that microbiome configurations were distributed along a continuous ecological gradient rather than forming clearly separated clusters.

Distance from the reference core tended to decrease as resilience increased, indicating that structurally stable microbiomes remained closer to the reference configuration. By contrast, MIS showed only a weaker association with compositional distance. Resilience therefore appeared more strongly associated with ecological proximity to the reference configuration than microbiological inflammatory pressure alone.

**Figure 4:**
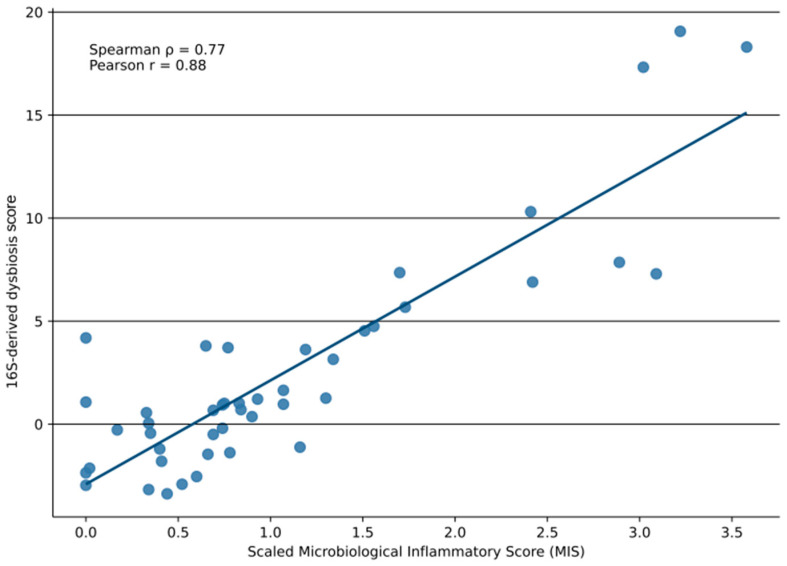
Relationship between MIS and the 16S-derived dysbiosis score. The dysbiosis index was calculated from the balance between protective taxa and pathobiont-associated taxa. The positive but imperfect association suggests that MIS and the dysbiosis index capture overlapping yet distinct aspects of microbiome imbalance. Pearson and Spearman correlation coefficients are shown to provide both linear and non-parametric evaluation of the observed ecological association. Correlation coefficients are provided for exploratory ecological interpretation and should not be interpreted as evidence of causality.

A positive but imperfect relationship was observed between MIS and the 16S-derived dysbiosis score derived from canonical dysbiosis-associated taxa (Figure 4). Linear regression analysis showed a strong positive association between MIS and the 16S-derived dysbiosis score (R^2^ = 0.78, Pearson r = 0.88, Spearman ρ = 0.77; *p* < 0.001). Microbiomes with elevated MIS values generally showed higher dysbiosis index values, whereas microbiomes with low MIS values tended to display lower or negative index values. The partial overlap between these metrics suggests that MIS and the dysbiosis index capture related but not identical dimensions of microbiome imbalance.

Similar discrepancies between global ecological descriptors and taxon-based dysbiosis indices have been reported in microbiome biomarker studies [7,25]. Because the present analysis is cross-sectional, these configurations should be interpreted as ecological states rather than temporal dynamics. Longitudinal studies will be required to determine the stability or transition dynamics between the different configurations identified within the MIS–MRS framework.

## 4. Discussion

We propose an exploratory framework integrating biological inflammation, microbiological inflammatory pressure, microbiome resilience, beta diversity, and a microbiome-derived metric for interpretation of canine gut microbiome profiles. The objective was to move beyond isolated taxonomic descriptions toward a multidimensional ecological interpretation of canine microbiome organization.

One important observation in the present cohort is that inflammation and microbiome imbalance do not always evolve in parallel. This partial dissociation has been noted before in canine enteropathies and helps explain why simple taxonomic readouts can be misleading in veterinary practice. Dog 9 showed concordance between biological inflammation and dysbiosis, combining high calprotectin, elevated MIS, increased ecological distance from the reference core, and low resilience. By contrast, Dogs 44, 15, and 41 displayed structurally stable microbiomes characterized by low inflammatory pressure and high resilience. Intermediate or transitional ecological states were represented by Dogs 34, 40, and 5, whereas Dog 4 illustrated a structurally fragile microbiome despite limited biological inflammatory activation. These representative configurations illustrate that inflammatory-associated microbial patterns, microbial resilience, and biological inflammation represent complementary dimensions of microbiome status.

The association observed between alpha diversity and resilience supports the biological relevance of the proposed framework. More diverse communities tended to be more resilient and clustered near the cohort’s reference core, while simplified communities were generally more fragile. Dogs 15, 41, and 44 are exemplifying high diversity paired with low inflammatory pressure and strong resilience. This observation is consistent with ecological theory linking diversity and functional redundancy to improved ecosystem stability. The ecological correlations observed further support the concept that dysbiosis in dogs may reflect a progressive ecological shift characterized by reduced resilience and decreased microbial diversity rather than a binary healthy/dysbiotic state. The moderate correlation strengths observed in this cohort are biologically plausible given the ecological heterogeneity of privately owned dogs and the multifactorial nature of intestinal inflammation. Interestingly, no strong direct association was observed between fecal calprotectin concentrations and dysbiosis score values, suggesting that inflammatory burden and ecological disruption may represent partially distinct biological dimensions.

However, diversity alone provides only a partial representation of microbiome organization. Microbiomes with similar Shannon diversity occasionally displayed markedly different resilience scores because of differences in functional composition or dominance structure. This highlights one of the limitations of relying solely on alpha diversity when interpreting canine microbiome data.

The moderate correlation observed between the reconstructed 16S-derived dysbiosis score and MIS further supports the multidimensional nature of microbiome organization. Although both variables were associated with dysbiotic configurations, they did not fully overlap, suggesting that compositional dysbiosis and inflammation-associated microbial patterns may represent partially distinct ecological processes.

To account for abundance-dependent ecological effects, the framework incorporated operational dominance rules in which the interpretative contribution of specific taxa varied according to their relative abundance within the microbial community. Taxa such as Megamonas, Fusobacterium, Proteus, and Enterobacteriaceae were therefore interpreted contextually rather than systematically classified as beneficial or detrimental, independently of ecological structure.

The Microbiological Inflammatory Score (MIS) showed a heterogeneous distribution across the cohort, with most dogs being under a low or mild inflammatory pressure, and a subset of dogs having MIS values consistent with dysbiosis-associated configurations. The weighting system and dominance thresholds used in MIS appeared operationally coherent within the present cohort. These findings support the concept that the MIS provides a broader ecological interpretation of community structure. Its correlation with the dysbiosis score supports its ecological relevance. While the MIS may be useful for analyzing real-world microbiome samples, it should be employed as an interpretative microbiome metric rather than as a clinically validated biomarker for decision-making.

Including resilience as a dimension reveals functional vulnerabilities that inflammatory metrics may miss. For instance, Dog 4 illustrated a structurally fragile microbiome despite limited biological inflammatory activation. Supporting the complementary value of inflammatory pressure and microbiome resilience for microbiome assessment. Beta diversity analysis reinforced the model’s internal coherence, showing that fragile communities occupy distinct compositional spaces separate from the reference core of low-inflammatory, high-resilience reference core.

These observations suggest that the MIS and resilience gradients reflect biologically coherent community reorganization rather than abstract constructs. Instead of attempting to define a universal “normal microbiome”, the present work uses an empirically defined reference core derived from microbiomes associated with low inflammatory pressure and preserved ecological stability.

All ecological analyses were based on relative abundance data derived from 16S rRNA sequencing. Because microbiome sequencing data are compositional by nature, relative abundance variations may not directly reflect absolute bacterial abundance changes and should therefore be interpreted cautiously. Nevertheless, relative abundance-based ecological metrics and Bray–Curtis dissimilarity remain widely used approaches in comparative microbiome ecology and were considered appropriate for the exploratory ecosystem-level objectives of the present study.

Microbiome configurations are therefore interpreted relative to their ecological distance from this reference centroid rather than against a predefined taxonomic composition. This approach also accommodates ecological heterogeneity, including variations in diet, lifestyle, housing environment, and supplementation, which are conditions under which veterinarians routinely analyze microbiomes. This framework may facilitate future evaluations of microbiota-modulating strategies such as dietary modulation, prebiotic or probiotic approaches.

Several limitations should be acknowledged. First, the cohort size remained moderate, with only a limited number of dogs presenting high inflammatory activity. Consequently, subgroup comparisons should be interpreted cautiously and cannot be considered definitive diagnostic validation. Second, dogs were recruited under real-life ecological conditions, resulting in substantial heterogeneity regarding diet, breed, age, supplementation, and environmental exposure. Although this heterogeneity reflects routine veterinary conditions, these variables may independently influence microbiome structure and resilience. Finally, because the present framework was developed operationally within the current cohort, external validation in larger and longitudinal canine populations will be necessary to evaluate reproducibility, threshold stability, and broader applicability.

In summary, the canine intestinal microbiome may be interpreted as a dynamic ecological system integrating biological inflammation, microbiological inflammatory pressure, structural resilience, beta diversity, and a dysbiosis-related metrics. This multidimensional approach may facilitate identification of both established dysbiosis and intermediate ecological configurations in canine microbiome research.

## 5. Conclusions

The framework proposed in this study integrates biological inflammation, microbiological inflammatory pressure, microbiome resilience, compositional dysbiosis, beta diversity, and a 16S-derived dysbiosis score into a unified ecological interpretation of the canine gut microbiome. Rather than establishing rigid diagnostic categories, this approach positions microbiomes along ecological gradients ranging from structurally stable communities to inflammatory dysbiosis. The combined use of calprotectin, MIS, MRS, beta diversity, and the dysbiosis score therefore provides a more integrative ecological interpretation than isolated microbiome descriptors alone.

Within this framework, microbiome profiles can be interpreted relative to al reference ecological core representing structurally stable microbial communities within the cohort.

The framework should primarily be viewed as an interpretative ecological tool designed for exploratory microbiome analysis in real-world veterinary datasets.

Future studies involving larger and clinically stratified cohorts together with longitudinal follow-up will be necessary to refine ecological thresholds and evaluate the predictive value of the MIS–MRS framework. Such studies could also help determine whether microbiome configurations identified within this framework are associated with clinical evolution or response to microbiota-modulating interventions.

## Figures and Tables

**Table 1:** Ecological Metrics of the Canine Cohort.

Dog	CalP (µg/g)	Shannon	Richness	Pielou	MIS_Scaled	MRS	16S DI	Bray–Curtis
1	6.250	2.08	22	0.67	1.70	0.78	7.354	0.658
2	0.000	2.13	20	0.71	2.89	0.75	7.854	0.904
3	15.950	2.85	35	0.80	2.42	0.85	6.896	0.590
4	6.250	1.04	7	0.53	3.22	0.58	19.066	0.951
5	19.150	2.49	23	0.79	1.73	0.82	5.677	0.654
6	27.750	2.72	31	0.79	3.58	0.84	18.303	0.678
7	6.250	2.47	29	0.73	1.51	0.80	4.529	0.589
8	14.400	1.94	28	0.58	2.41	0.69	10.311	0.738
9	78.950	1.35	10	0.59	3.02	0.33	17.323	0.923
10	6.250	2.36	24	0.74	3.09	0.79	7.295	0.655
11	6.250	2.49	19	0.84	0.661	0.83	−1.461	0.739
12	6.250	2.91	31	0.85	0.328	0.88	0.556	0.795
13	6.250	2.48	18	0.86	0.521	0.84	−2.915	0.765
14	6.250	2.75	20	0.92	0.020	0.88	−2.140	0.617
15	6.250	3.52	53	0.89	0.41	0.96	−1.801	0.455
16	57.600	3.14	29	0.93	1.07	0.93	0.967	0.606
17	12.900	2.87	22	0.93	0.69	0.90	0.673	0.486
18	6.250	2.84	23	0.91	0.77	0.89	3.713	0.720
19	6.250	2.85	27	0.86	1.16	0.88	−1.117	0.521
20	37.750	3.51	52	0.89	0.34	0.96	0.043	0.654
21	14.400	2.38	20	0.79	0.65	0.81	3.799	0.818
22	12.550	3.13	38	0.86	0.17	0.91	−0.278	0.427
23	30.700	1.91	12	0.77	0.74	0.74	−0.197	0.803
24	6.250	1.40	6	0.78	0.00	0.69	4.186	0.903
25	14.050	3.32	41	0.89	0.93	0.94	1.216	0.460
26	16.350	3.17	36	0.89	0.00	0.92	−2.967	0.673
27	12.500	3.13	34	0.89	0.44	0.92	−3.380	0.554
28	6.250	3.43	55	0.86	0.74	0.94	0.936	0.558
29	6.250	3.20	34	0.91	0.90	0.93	0.364	0.475
30	13.600	3.17	32	0.91	0.78	0.93	−1.385	0.656
31	15.700	3.28	33	0.94	1.07	0.95	1.642	0.424
32	60.650	3.40	49	0.87	1.34	0.94	3.147	0.646
33	14.700	2.84	22	0.92	0.83	0.89	1.021	0.557
34	19.000	2.30	13	0.90	0.60	0.83	−2.543	0.727
35	6.250	2.92	30	0.86	1.19	0.89	3.622	0.618
36	6.250	2.65	21	0.87	0.00	0.86	−2.362	0.552
37	29.300	2.14	24	0.67	0.00	0.74	1.070	0.904
38	6.250	3.31	41	0.89	0.69	0.94	−0.498	0.504
39	6.250	2.89	27	0.88	1.30	0.89	1.262	0.585
40	15.700	1.97	14	0.75	0.84	0.75	0.705	0.885
41	13.800	2.91	27	0.88	0.34	0.89	−3.174	0.546
42	22.450	2.95	27	0.89	0.40	0.90	−1.202	0.551
43	82.500	3.01	36	0.84	0.75	0.89	1.007	0.625
44	6.250	3.56	50	0.91	0.35	0.97	−0.438	0.372
45	6.250	2.22	15	0.82	1.56	0.80	4.749	0.633

Main analytical cohort (n = 45). Two additional 16S profiles (SampleID 202505-025 and 202505-00E) were retained only for comparative sequencing purposes and were excluded from the integrated ecological analysis because of insufficient analytical quality for calprotectin measurement. Abbreviations: CalP, fecal calprotectin; MIS, Microbiological Inflammatory Score; MRS, Microbiome Resilience Score; 16S DI, 16S-derived dysbiosis score.

## Data Availability

The datasets generated and analyzed during the current study are available from the corresponding author upon reasonable request. Access may be subject to restrictions related to ongoing research, confidentiality, or institutional policies.

## Supplementary Materials

The following supporting information can be downloaded at: https://www.mdpi.com/article/10.3390/ani16121787/s1. File S1: Detailed methodological description of the ecological interpretation framework, including MIS calculation, MRS calculation, alpha and beta diversity metrics, reference core microbiome construction, 16S-derived dysbiosis index, and integrative ecological interpretation procedures.
