# Supplementary material for: An Ecological Framework for Interpreting the Canine Gut Microbiome
Source: Animals (Basel). 2026 Jun 9;16(12):1787. doi: 10.3390/ani16121787 (PMC13295516; doi:10.3390/ani16121787)
Supplement: Supplementary file 1 [file animals-16-01787-s001.zip › Supplementary File S1.pdf]

Supplementary Material and Methods – Detailed Framework

Analytical Framework for Ecological Interpretation of the Canine Gut Microbiome

This document provides a detailed description of the analytical framework used to interpret canine gut microbiome profiles in the present study. The framework integrates multiple complementary ecological descriptors, including microbiological inflammatory pressure, microbiome resilience, alpha diversity, beta diversity, and a 16S-derived dysbiosis index. Together, these parameters allow microbiomes to be interpreted within an ecological continuum ranging from structurally stable microbial ecosystems to inflammatory dysbiosis.

S1. Microbiological Inflammatory Score (MIS)

The Microbiological Inflammatory Score (MIS) was developed to estimate the inflammatory pressure exerted by the microbiome based on taxonomic composition and abundance.

The raw MIS was calculated as a weighted sum of the relative abundances of selected bacterial taxa:

$MIS_{raw} = \sum_{i=1}^{S}(A_i \times W_i)$MISraw=i=1∑S(Ai×Wi)

where:

- A_iAi represents the relative abundance of taxon ii
- W_iWi represents the taxonomic weight assigned to taxon ii
- SS represents the number of taxa included in the calculation

Taxonomic weights were defined based on current literature describing associations between bacterial taxa and inflammatory processes in the canine gut microbiome. Positive weights were assigned to taxa associated with inflammatory or dysbiotic configurations, whereas negative weights were assigned to taxa generally associated with microbial stability or beneficial metabolic functions.

Because microbial dominance can amplify ecological effects, a dominance adjustment was applied:

$MIS_{adj} = MIS_{raw} \times (1 + Dominance\ factor)$MISadj=MISraw×(1+Dominance factor)

Finally, the adjusted score was normalized to produce the scaled MIS used in the analyses:

$MIS_{scaled} = \frac{MIS_{adj}}{Scaling\ constant}$MISscaled=Scaling constantMISadj

The MIS should be interpreted as an ecological indicator of microbiological inflammatory pressure rather than as a clinically validated diagnostic biomarker.

S2. Microbiome Resilience Score (MRS)

The Microbiome Resilience Score (MRS) was designed to estimate the structural stability of the microbial ecosystem by integrating diversity, evenness, and functional balance.

The score was calculated as:

$MRS = \frac{H \times J \times Functional\ Balance}{1 + Dominance\ factor}$MRS=1+Dominance factorH×J×Functional Balance

where:

- HH represents the Shannon diversity index
- JJ represents Pielou evenness
- Functional Balance represents the ratio between taxa associated with beneficial microbial functions and taxa associated with inflammatory pressure.

This formulation reflects ecological principles whereby microbial ecosystems with higher diversity and balanced functional composition tend to exhibit greater resilience to perturbation.

The MRS therefore represents an ecological indicator of microbiome robustness and structural stability.

S3. Alpha Diversity

Alpha diversity describes the diversity and internal structure of microbial communities.

Shannon diversity index

$H = -\sum_{i=1}^{S}(p_i ln\ p_i)$H=−i=1∑S(pilnpi)

where:

- $p_i$pi represents the relative abundance of taxon ii
- SS represents species richness.

The Shannon index accounts for both species richness and evenness.

Pielou evenness

$J = \frac{H}{ln\ S}$J=lnSH

where:

- HH represents Shannon diversity
- SS represents species richness.

Pielou evenness ranges from 0 to 1 and describes how evenly taxa are distributed within the community.

S4. Beta Diversity and Reference Core Centroid

Beta diversity between microbiome profiles was quantified using Bray–Curtis dissimilarity.

$BC(x,y) = 1 - \frac{2\sum_{i=1}^{S} min\,(x_i,y_i)}{\sum_{i=1}^{S} x_i+\sum_{i=1}^{S} y_i}$BC(x,y)=1−∑i=1Sxi+∑i=1Syi2∑i=1Smin(xi,yi)

where:

- $x_i$xi and $y_i$yi represent the relative abundance of taxon ii in microbiomes xx and yy
- SS represents the total number of taxa included in the analysis.

Bray–Curtis dissimilarity ranges from **0 (identical microbiomes)** to **1 (completely different communities)**.

Reference core microbiome selection

To provide an ecological reference point for interpreting microbiome variation, a subset of microbiomes representing structurally balanced microbial ecosystems was identified within the cohort.

Selection criteria included:

- low fecal calprotectin concentrations
- low microbiological inflammatory pressure (low MIS values)
- relatively high microbiome resilience and alpha diversity
- absence of strong dominance by taxa associated with inflammatory states.

These microbiomes were considered representative of **ecologically coherent microbiome configurations**.

Reference centroid construction

The reference centroid represents the average microbial composition of the selected core microbiomes.

For each taxon ii, the centroid abundance was calculated as the mean relative abundance across the selected microbiomes:

$Centroid_i = \frac{1}{n}\sum_{k=1}^{n} A_{ik}$Centroidi=n1k=1∑nAik

where:

- A_{ik}Aik represents the relative abundance of taxon ii in microbiome kk
- nn represents the number of microbiomes included in the reference core.

The resulting vector of average abundances defines the **reference microbiome centroid**, which serves as a structural reference configuration for balanced microbial ecosystems.

Distance to the reference core
For each microbiome sample, the compositional distance to the reference centroid was calculated using Bray–Curtis dissimilarity:

$D_{core} = BC(sample, centroid)$Dcore=BC(sample,centroid)

where:

- *sample* represents the microbiome being evaluated
- *centroid* represents the reference core composition.

Lower values indicate microbiomes structurally similar to the reference ecosystem, whereas higher values reflect increasing compositional deviation.

S5. Dysbiosis Index (16S-derived)
The dysbiosis index was derived from 16S sequencing data and reflects the balance between taxa associated with protective microbial functions and taxa associated with inflammatory or dysbiotic states.

The index was calculated as:

$DI = log_{10}\left(\frac{\sum weighted\ pathobionts}{\sum weighted\ beneficial\ taxa}\right)$DI=log10
(∑weighted beneficial taxa∑weighted pathobionts)

This formulation provides an integrated representation of microbiome imbalance.

Unlike previously described qPCR-based dysbiosis indices, the 16S-derived index used in the present study captures a broader ecological representation of microbial community structure.

S6. Integrative Ecological Interpretation Framework
The analytical framework combines:

- microbiological inflammatory pressure (MIS)
- microbiome resilience (MRS)

- alpha diversity
- beta diversity
- distance to a reference microbiome centroid
- dysbiosis index

Together, these parameters allow microbiome profiles to be positioned along a continuum ranging from structurally stable microbial ecosystems to inflammatory dysbiosis.

Within this framework, the reference centroid serves as an ecological anchor allowing microbiomes to be interpreted relative to balanced microbial configurations rather than solely relative to cohort averages.

The derived metrics are intended as **interpretative ecological indicators** and not as standalone clinical diagnostic biomarkers. The framework should therefore be considered an exploratory ecological interpretation tool rather than a diagnostic classification system.
